# Multiple influence of immune cells in the bone metastatic cancer microenvironment on tumors

**DOI:** 10.3389/fimmu.2024.1335366

**Published:** 2024-02-23

**Authors:** Shixin Chen, Jiangchu Lei, Haochen Mou, Wenkan Zhang, Lingxiao Jin, Senxu Lu, Eloy Yinwang, Yucheng Xue, Zhenxuan Shao, Tao Chen, Fangqian Wang, Shenzhi Zhao, Xupeng Chai, Zenan Wang, Jiahao Zhang, Zengjie Zhang, Zhaoming Ye, Binghao Li

**Affiliations:** ^1^ Department of Orthopedic Surgery, The Second Affiliated Hospital, Zhejiang University School of Medicine, Hangzhou, China; ^2^ Orthopedics Research Institute of Zhejiang University, Hangzhou, Zhejiang, China; ^3^ Key Laboratory of Motor System Disease Research and Precision Therapy of Zhejiang Province, Hangzhou, Zhejiang, China; ^4^ Clinical Research Center of Motor System Disease of Zhejiang Province, Hangzhou, Zhejiang, China

**Keywords:** bone metastatic cancer, tumor immune microenvironment, TME, immune cell, immunosuppression, metastasizing

## Abstract

Bone is a common organ for solid tumor metastasis. Malignant bone tumor becomes insensitive to systemic therapy after colonization, followed by poor prognosis and high relapse rate. Immune and bone cells *in situ* constitute a unique immune microenvironment, which plays a crucial role in the context of bone metastasis. This review firstly focuses on lymphatic cells in bone metastatic cancer, including their function in tumor dissemination, invasion, growth and possible cytotoxicity-induced eradication. Subsequently, we examine myeloid cells, namely macrophages, myeloid-derived suppressor cells, dendritic cells, and megakaryocytes, evaluating their interaction with cytotoxic T lymphocytes and contribution to bone metastasis. As important components of skeletal tissue, osteoclasts and osteoblasts derived from bone marrow stromal cells, engaging in ‘vicious cycle’ accelerate osteolytic bone metastasis. We also explain the concept tumor dormancy and investigate underlying role of immune microenvironment on it. Additionally, a thorough review of emerging treatments for bone metastatic malignancy in clinical research, especially immunotherapy, is presented, indicating current challenges and opportunities in research and development of bone metastasis therapies.

## 1 Introduction

Bone metastasis is a common target organ of metastasis for several solid tumor types, including lung, breast, prostate, colorectal, thyroid, and gynecological tumors, and melanoma. Statistically, approximately 70% of patients with metastatic prostate and breast cancer develop bone metastases (1). Once cancer has spread to the bone, it is usually difficult to cure and is accompanied by a variety of accompanying complications such as pain, increased risk of fractures, and hypercalcemia (2).

Early studies of bone metastatic cancer present a phenomenon known as the “vicious cycle,” in which interactions between tumor cells and bone cells exacerbate the development of bone metastatic cancer (3). Tumor cells release substances such as parathyroid hormone-related protein (PTHrP), which stimulates osteoblasts to produce nuclear factor B receptor-activated ligand (RANKL), which further activates osteoclasts and leads to osteolysis. In turn, the multiple factors produced by osteolysis further promote tumor growth and more bone loss (4). This study reveals the impact of the bone microenvironment on the interactions between tumor cells.

The tumor microenvironment (TME) is a composite of various components (5), including the immune microenvironment. Meanwhile, the bone plays an important role as an immune organ in the body. The immune microenvironment of bone metastatic cancer is characterized by immune cells, such as T cells, macrophages, dendritic cells (DC), megakaryocytes, and myeloid-derived suppressor cells (MDSCs) (6). MDSCs are derived from immature myeloid progenitor cells and inhibit the immune function of T cells and NK cells in the TME (7). In addition, the bone microenvironment contains two key cell types: osteoblasts and osteoclasts. Osteoblasts are derived from multiple potential mesenchymal stem cells in the bone marrow stroma (8). Most prostate cancer bone metastases are osteogenic, with tumor cells tending to promote osteogenic activation of osteoblasts, whereas osteoclasts are derived from monocytes and are responsible for bone resorption (9). In osteolytic tumors such as bone metastases of the breast, lung, and kidney, tumor cells tend to promote the osteolytic function of osteoclasts.

Key factors in the immune microenvironment may include the local cytokine environment, the presence of helper stromal cells, specific types of immune cells, all of which play an important role in tumor-specific interactions (10). Different types of immune cells exert different functions in the immune microenvironment of metastatic bone cancer. Immune cells, such as NK cells and cytotoxic T cells, are capable of directly killing tumor cells using different mechanisms, whereas other immune cell subtypes, such as regulatory T cells (Tregs), a subtypes of CD4+ T helper cells, M2-type macrophages, tolerogenic DC, and MDSCs, inhibit adaptive immune responses to tumors, thereby promoting tumor progression and metastasis (11).

In metastatic cancer of the bone, specific cells in the bone and immune cells share a common environment (12), therefore this paper reviews the different types of cells and their effects in the immune microenvironment, and discusses areas for future development in this field.

## 2 Lymphatic immune cells

### 2.1 T cells

T cells originate from hematopoietic stem cells and lymphoid progenitors stored in the bone marrow and differentiate into primary lymphoid organs waiting to be activated by antigens. Partial tumor-infiltrating lymphocytes (TIL) in tumors have tumor cell-killing function (13). However, in the immunosuppressive microenvironment of bone metastatic cancer, T cells can be suppressed, leading to depletion or inactivation of T cells (14). Furthermore, the microenvironment of bone metastatic cancer also attracts other T cell subtypes, such as Tregs and other CD4+ T cells, which can support tumor growth and metastasis (11). For example, T cells can promote the development and maturation of osteoclasts, which can lead to the malignant cycle of bone metastatic cancer. In bone metastatic cancer, T cells are recruited and activated by tumor secreted factors such as PTHrP, interleukin (IL)-7, and IL-8, and recruited T cells can secrete tumor necrosis factor-α (TNF-α) or RANKL to induce bone resorption (15), which allows T cells to also participate in the vicious cycle process. Of course, this osteoclast-promoting effect is unique to nonactivated T cells (16).

T cells can be classified according to their function as cytotoxic T lymphocytes (CTL) and helper T cells (Th), where Th cells can be further classified as Th1, Th2, Th9, Th17, Th22, Tfh, and Tregs, depending on their function. Recent studies have shown that Th1, Th2, Th17, and Tregs are involved in the occurrence and development of tumor cells in bone metastases (17–21).

#### 2.1.1 Cytotoxic T lymphocytes

CTLs are closely associated with the anticancer immune response, as these cells expresses a CD8 glycoprotein on its surface, and are also known as CD8+ T cells (22). They are activated to kill tumor cells by interacting with DC that present tumor-specific antigens (23).

Interferon-γ (IFN-γ) produced by CTL plays a key role in determining the antitumor ability of CTLs. Production of IFN-γ contributes to the tumor expression of MHC-I, making them more easily recognizable by CTLs (24), and directly inhibits tumor cell proliferation and induces apoptosis, thus exerts a direct role in the fight against cancer (25). IFN-γ from other sources also plays an important role in CTLs. PD-L1 deficiency in myeloid cells in bone metastatic cancers upregulates immunostimulatory genes, thereby contributing to macrophage polarization towards the M1 type and enhances IFN-γ signaling, which promotes the recruitment and activation of CTLs (26). Additionally, the presence of IFN-γ reduces tumor-associated bone loss and inhibits osteoblast development (27). However, IFN-γ also inhibits tumor cell killing by immune cells. Specifically, IFN-γ inhibits CTL function by upregulating the expression of programmed death ligand 1 (PD-L1) on the surface of tumor cells; thus, increasing its binding to the programmed cell death-1 (PD-1) receptor on the surface of CTL cells (28). IFN-γ can also activate interferon regulatory factor 2 (IRF2), a CTL transcription factor in the TME, thus changing CTL from an activated state to a depleted state and forcing tumor cells to evade immune surveillance (29).

CTLs are also affected by osteoclasts in bone metastatic cancer. Osteoclasts have been shown to undergo apoptosis and produce apoptotic vesicles during cyclic bone remodeling in bone metastatic cancer, thus inhibiting the infiltration and activity of CTLS (30). Conversely, osteoclasts exert a positive regulatory effect on CTLs. Lyn(-/-) mice, with more numerous osteoclasts, had reduced bone tumor growth despite enhanced osteolysis, due to the increased tumor-killing function of CTLs as a result of the increase in osteoclasts (31).

Furthermore, in addition to playing an important role in tumor metastasis to bone, CTLs also exert multiple critical functions in different types of bone metastatic cancers. Overexpression of estrogen-related receptor alpha (ERRα) in breast cancer bone metastases activates the tumor-killing effect of CTLs, through the production of chemokines C-C chemokine receptor type 17 (CCL17) and C-C chemokine receptor type 20 (CCL20), which allows CTLs to evade the control of transforming growth factor-β (TGF-β) (32). The up-regulation of IL-27 in bone metastasis of prostate cancer can lead to up-regulation of genes related to T-cell activation (33). The signal transducer and activator of the transcription 6 (STAT6) pathway is important in CTL immune suppression, which can be independent of Tregs (34). Snail(+) tumor cells can secrete Human follistatin-like protein 1 (FSTL1) not only to directly promote tumor bone metastasis, but also to generate CD45(-) activated leukocyte cell adhesion molecule (ALCAM)(+) cells, which can be surrounded by CD8+ T cells with weak CTL activity that contribute to the development of bone metastatic cancer (35).

#### 2.1.2 Th cells

**Figure 1:**
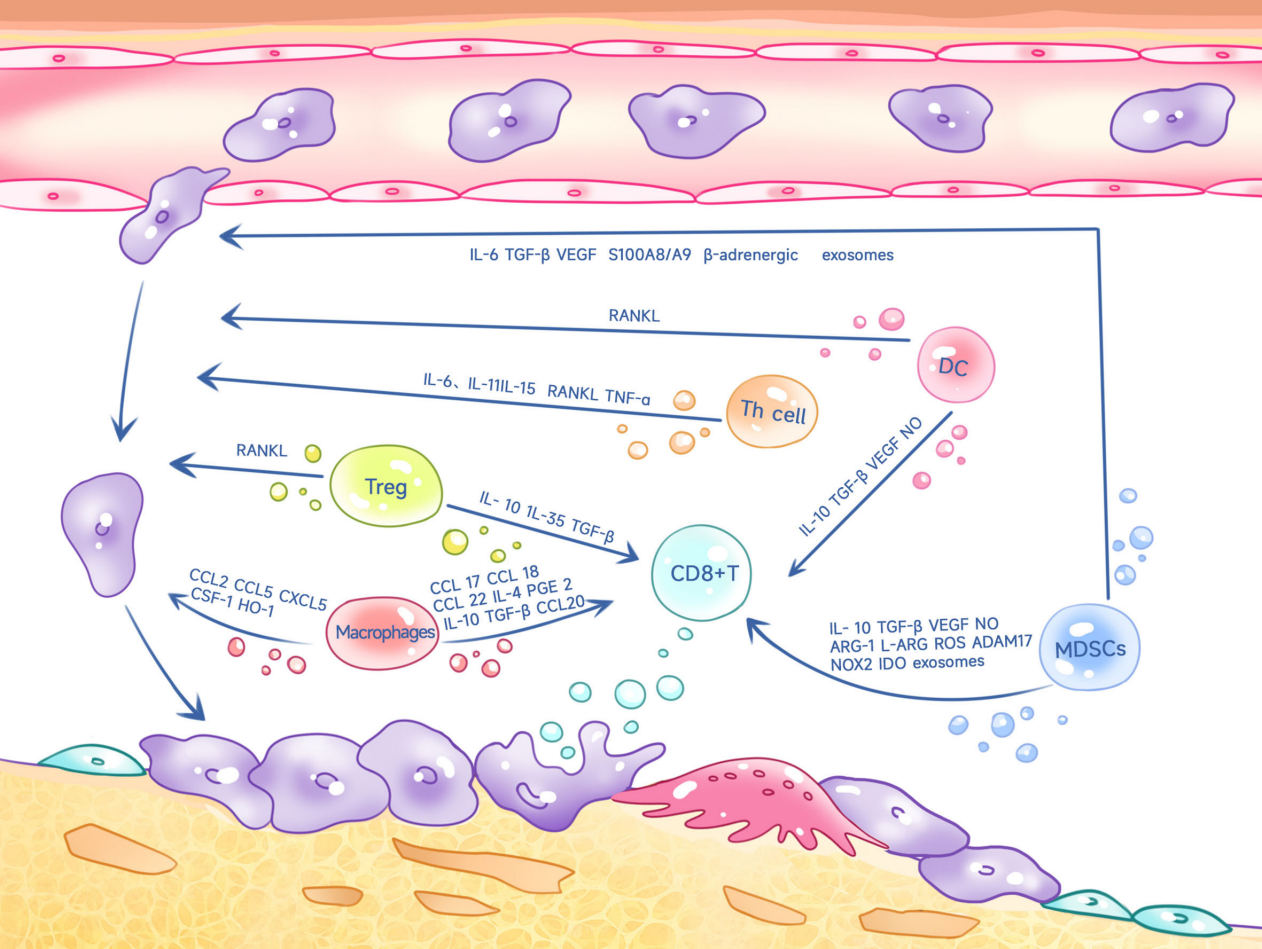
Th cells, Tregs, macrophages, MDSCs, DC cells inhibit CD8+ T cell killing on tumors and promote tumor cell metastasis to bone through multiple factors.

Th cells are immune T cells that produce cytokines involved in the adaptive immune response (36). Th cells play an important role in the mechanisms regulating of entry tumors into the bone environment and subsequent adaptive immune processes. Activated Th cells also release a variety of factors, such as IL-6, IL-11, IL-15, RANKL, and TNF-α, which promote osteoclastogenesis and bone resorption and provide a favorable environment for tumor bone metastasis (37, 38) (**Figure 1**). IL-7 is involved in T-cell proliferation and activation and can prompt CD8+ and CD4+ T cells to produce factors such as RANKL and TNF-α (39). These factors play a role in bone metastasis in the bone environment. However, activated CD4+ T cells also produce IFN-γ, which can inhibit osteoclast activity (40). Thus, Th cells influence the bone environment by releasing multiple factors and are also involved in regulating immune infiltration of tumors. Some studies have also implicated Th cells in the process of bone metastasis (41). Of particular note, in breast cancer, inhibition of poly ADP-ribose polymerase 2 (PARP2) increases the risk of bone metastasis, as this leads to an increase in immature myeloid cells in the bone marrow, which inhibits Th cell recruitment and creates an immunosuppressive microenvironment (42).

As mentioned previously, naïve Th cells can differentiate into different subtypes, including Th1, Th2, Th17, and Tregs, which exert different in the tumor immune response. This further highlights the importance of Th cells in bone metastatic cancer. Th1 cells in bone metastases of melanoma are affected by intestinal microbes, and when intestinal microbes are depleted, Th1 cell growth is inhibited, accelerating tumor growth and osteolysis (17). Despite the increase in CD4+ T cells within prostate cancer bone metastases, they are insensitive to immune checkpoint therapy, because CD4+ T cells differentiate into the Th17 rather than the Th1 line, mechanically because bone tumors promote osteoclast-mediated bone resorption, releasing TGF-β, which inhibits the development of the Th1 line (18). Wang et al. used genetically engineered hematopoietic stem cells (HSC) to deliver a small molecule inhibitor of TGF-β to the bone marrow, which resulted in the differentiation of CD4+T into Th1 and Th2 cells (19). LysM(Cre)/Tgfbr2 knockdown significantly inhibits the proliferation, angiogenesis, and osteoclast activation of metastatic cancer (43).

#### 2.1.3 Tregs

Regulatory T cells (Tregs) are an important subpopulation of CD4+ T cells that are essential for the induction and maintenance of normal peripheral tolerance and the prevention of autoimmunity (20). Tregs can inhibit the function of many cells in bone metastatic cancers, including CD8+ T cells and Th1 cells, thus generating an immunosuppressive microenvironment (21). The specific mechanism is exerted through IL-10, IL-35, and TGF-β activity (44, 45) (**Figure 1**). Additionally, Tregs significantly inhibited the proliferation of CD4+CD25- T cells by directly contacting and thus blocking the delivery of costimulatory signals (46).

Increased Treg infiltration in prostate cancer often leads to a poor prognosis. In the immune microenvironment of bone metastasis from prostate cancer, Tregs can translocate to the bone marrow through C-X-C chemokine receptor 4 (CXCR4)/C-X-C Motif Chemokine 12 (CXCL12) (47). In addition to its immunosuppressive effects, Forkhead box protein P3 (Foxp3)+ Tregs are a key source of RANKL (48). As mentioned previously, RANKL produced by T cells can promote osteolysis to bone metastasis (37) (**Figure 1**). Bone marrow DC, in turn, promote the proliferation of Tregs through the receptor activator of NF-KappaB (RANK)-RANKL axis (47, 49). Thus, Tregs form a positive feedback axis for tumor bone metastasis and osteolysis through the RANK-RANKL axis.

Expression of CD73 on the surface of Tregs can also promote tumor metastasis (50). During the immune response, Th17 cells can be transformed into Tregs, which results from activation of the aromatic hydrocarbon receptor (AHR) by TGF-β (51). Activation of TNFR2 in Tregs by TNF can promote the expansion of immunosuppressive Tregs (52). The Tregs bone metastatic cancer microenvironment also interacts with osteoblasts and osteoclasts. For instance, osteoblasts can inhibit the function of CTLs by creating a suitable environment for Tregs through aerobic glycolysis (53). Tregs regulate osteoclast differentiation through cytotoxic T lymphocyte antigen (CTLA-4) in cell-to-cell contacts (54), thus altering the immune microenvironment of bone metastatic cancer.

### 2.2 Natural killer cells

Natural killer cells (NK cells) rapidly recognize and destroy cancer cells (55). For example, NK cells can release perforin and granzyme, mechanisms that lead to apoptosis of cancer cells (56).

However, several factors can influence the function of NK cells. In the early stages of bone metastasis, estrogen receptor (ER)-positive luminal cancers release signal peptide, CUB domain and EGF-like domain containing 2 (SCUBE2), which may help to induce osteoblasts to differentiate into osteoclasts, thereby inhibiting NK cells activity and providing favorable conditions for tumor colonization (57). In breast cancer, overactivation of the Janus kinase (JAK)/STAT signaling pathway has been widely reported (58, 59). However, inhibition of the JAK/STAT signaling pathway decreases the antitumor immune function of NK cells in metastatic tumors (60). Gut microbial deprivation also inhibits the proliferation of NK cells in bone metastatic tumors (17). This phenomenon suggests that intestinal microbes may play a role in modulating the immune response. Furthermore, in the case of neuroblastoma bone metastases, IL-2 therapy has been shown to be effective, suppressing tumors by increasing NK cell activity (61). Overexpression of PTHrP promoted bone metastasis of small-cell lung cancer in a mouse model, which may be related to NK cell depletion, but the exact mechanism needs to be further investigated (62).

## 3 Myeloid immune cells

### 3.1 Macrophages

Macrophages are an important component of the mononuclear phagocyte system (MPS) and are key cells in the tumor immune system (63). The main immune cells that infiltrate tumors are macrophages, also known as tumor-associated macrophages (TAM) (64). There is growing evidence that these macrophages play an important role in the immune microenvironment of bone metastatic cancer and play a key regulatory role in tumor progression, angiogenesis, invasion, and metastasis (65–67).

#### 3.1.1 Impact on adaptive immunity

Macrophages can polarize into M1 and M2 types in the TME (68–70). M1 macrophages can produce reactive oxygen species (ROS), have high antigen presentation potential, and can recruit CTLs (71–73). Although M2 macrophages are usually considered pro-tumorigenic, M2 macrophages can recruit Tregs and Th2 cells by secreting anti-inflammatory factors that induce adaptive immune incompetence of the body against tumors (74, 75).

The chemokine CCL20 is highly expressed in macrophages, as is the homologous C–C chemokine receptor type 6 (CCR6) expressed on T cells. Macrophages in bone metastatic cancers inhibit the immune response of T cells to tumors through regulation of the CCL20-CCR6 axis (76). Furthermore, TAM-derived CCL17, CCL18, CCL22, IL-4, IL-10, TGF-β, and prostaglandin E2 (PGE 2) can inhibit the antitumor function of T cells (77–83). In bone metastases of prostate cancer, fusion of tumor cells with myeloid cells, including macrophages, further suppresses the immune response while promoting tumor growth (84) (**Figure 1**).

#### 3.1.2 Impact on tumor metastasis

Macrophages can promote the growth of tumor cells in bone in several ways (85), and inhibition of macrophages reduces the incidence of tumor bone metastasis (86). Targeting anti-CD115 antibodies reduces the number of macrophages in tumors and therefore reduces osteolytic lesions in transplanted breast cancer cells (87). The interaction between macrophages and prostate cancer cells contributes to upregulation of cathepsin K expression in macrophages, which promotes tumor progression within metastases (88). CD137, a member of the TNF receptor superfamily, can promote macrophage migration into the TME and stimulate macrophage transformation into osteoclasts by enhancing Fra1 expression, thus promoting tumor bone metastasis (89). Furthermore, CCL5 secreted by TAMs contributes to bone metastasis of prostate cancer (90). CCL2 can help prostate tumor growth and bone metastasis by recruiting macrophages and osteoclasts (91) and macrophages also promote breast cancer bone metastases in an IL-4R-dependent manner, and inhibition of IL-4R effectively reduces the occurrence of bone metastases (92). Furthermore, CXCL5 and colony stimulating factor 1 (CSF-1) are associated with macrophage-driven bone metastasis (87, 93–95) Cyclooxygenase-1 (COX-1) positive macrophages can play an important role in prostate cancer bone metastasis (96) (**Figure 1**).

These studies highlight the important role of macrophages in immunomodulation and bone metastasis and provide useful information to better understand the onset and progression of bone metastatic cancer.

### 3.2 Myeloid-derived suppressor cells

Myeloid-derived suppressor cells (MDSC) are distinct immunosuppressive cells in tumors. MDSCs consist of a heterogeneous population of immature myeloid cells (IMCs) with immunosuppressive functions (97). MDSCs have been found to be widely infiltrated in a wide variety of cancers and have a significant ability to suppress T cell responses, leading to a poor prognosis. Such cells have attracted increasing attention in the academic community (81). Furthermore, MDSCs play a crucial role in bone (98).

#### 3.2.1 Impact on adaptive immunity

MDSCs inhibit T cell proliferation by suppressing the immune response of T cells in several ways: (i) by generating arginase-1 (ARG-1)-dependent depletion and chelating L-cysteine depletion of L-arginine to inhibit T cell proliferation; (ii) by interfering with the signaling of the IL-2 receptor and generating ROS and NO to inhibit T-cell function; (iii) by expressing ADAM 17 (which contains structural domains of de-integrins and metalloproteinases17) and galactose lectin 9 that interfere with T-cell metastasis and pro-apoptosis activity; and (iv) by inducing Treg proliferation, which promotes bone metastasis growth (99–101). MDSCs also inhibit NK cell function via TGF-β, and IL-10 (102–104) inhibits dendritic cell differentiation and antigen presentation through IL-10, vascular endothelial growth factor (VEGF), NADPH oxidases (NOX2), and ROS (103, 105–108). Recent studies have shown that MDSC-produced exosomes can overactivate or deplete CD8+ T cells, thus suppressing immune function (109). In bone metastasis of breast cancer, MDSCs express PD-L1, which not only inhibits T cell function, but also promotes osteoclastogenesis, thus facilitating the progression of bone metastatic cancer (16) (**Figure 1**).

#### 3.2.2 Impact on tumor metastasis

In the metastatic bone cancer microenvironment, MDSCs not only have immunosuppressive effects, but also accelerate bone lysis and destruction. MDSCs can promote bone tumor metastasis through a variety of mechanisms (98, 110). Tumors recruit MDSCs through the CCL2/CCL12-CCR2, CCL3/4/5-CCR5, CCL15-CCR1, CX3CL1/CCL26-CX3CR2, CXCL5/CXCL2/CXCL1-CXCR2, CXCL8 (IL-8)-CXCR1/CXCR2, CCL21-CCR7, CXCL13-CXCR5 pathways, promoting immunosuppression in the TME, while, MDSCs also promote tumor metastasis via the CCL5/CCR5,CCL15-CCR1,CXCL5/CXCL1-CCR2, CXCL8 (IL-8)-CXCR1/CXCR2 pathways (111). MDSCs also secrete TGF-β, S100A8/A9, VEGF and exosomes to interact with the immune system, endothelial cells, fibroblasts, and liver stellate cells, thus making the bone microenvironment suitable for tumor implantation (112). Furthermore, in a 4T1 mouse metastasis model, inhibition of interferon regulatory factor 7 (IRF7) enhanced the prometastatic activity of MDSCs. Conversely, IRF7 overexpression can counteract the effects of MDSCs and restore the activity of CD8+ T cells and NK cells to reduce metastasis (113). MDSCs can also promote tumor metastasis by enhancing β-adrenergic signaling and the IL-6/STAT3 pathway (114). The prometastatic effects of MDSCs are also closely related to osteoclasts. MDSCs derived from bone metastatic cancers can be induced to become osteoclast progenitors and can differentiate into osteoclasts (115). In addition to becoming osteoclasts themselves, they can also induce osteoclastogenesis, and in bone metastatic cancers, MDSC-produced nitric oxide (NO) not only mediates immunosuppression, but also mediates osteoclast generation (98, 99). Due to the fact that bone metastasized tumors express hypoxia-inducible factor (HIF)-1α at a higher level than primary tumors, which plays an important role in osteoclast formation (98, 99), and NO in turn up-regulates HIF-1α through various mechanisms, such as phosphatidylinositol 3-kinase and schizogen-activated protein kinase (116). Tumor cell levels of PTHrP and GL I-Kruppel 2(Gli 2) can be induced by MDSCs, which are also involved in osteoclastogenesis (110) (**Figure 1**).

### 3.3 Dendritic cells

DC are a class of immune cells that originate in the bone marrow and are widely distributed in various tissues (117–119). They play a key role in the induction and regulation of innate and adaptive immune responses by antigen presentation (117–120). DC efficiently phagocytose apoptotic cells and cross-present viral, tumor, and autoantigens to CD8(+) T cells (121, 122). In tumors, the role of DC is crucial, as they are capable of initiating an effective T cell response, attracting T cells to the tumor site, and maintaining the function of effector memory T cells (22, 123). Circulating DC readily migrate to the bone marrow due to the high expression of vascular cell adhesion molecule-1 (VCAM-1) and endothelial selectin in the microvasculature of the bone marrow, which is critical for metastatic bone cancers (124).

#### 3.3.1 Impact on adaptive immunity

DC can differentiate into two subpopulations: myeloid DC (mDC) and plasmacytoid DC (pDC) (123). Although the importance of DC in antitumor immune responses is well known, cancer cells can still promote an immunosuppressive phenotype by affecting DC. In metastatic bone cancer, DC in the TME inhibit the tumor-killing activity of CD8+ T cells by producing cytokines such as IL-10, VEGF, TGF-β, and NO (125). IL-6 produced by tumor cells can contribute to the differentiation of hematopoietic stem and progenitor cells (HSPC) into monocyte-dendritic progenitor cells (MDPs) (126). Furthermore, high expression of CD1a(+) and CD83(+) has been reported to be negatively correlated with the development of bone metastases (127). In breast cancer bone metastases, pDC can persistently activate Th2, increase infiltration of Tregs and MDSCs, and produce osteolytic cytokines, leading to severe bone destruction (128). Mitochondrial transcription factor A (TFAM) deletion improves the presentation of antigens by DC through the cGAS-STING pathway, reversing immunosuppression in the TME (129). Furthermore, DC can induce the production of the PTHrP-derived peptide, PTR-4, which maintains CTL activation and thus improves tumor killing (130).

#### 3.3.2 Impact on tumor metastasis

DC also play a key role in promoting tumor metastasis. TGF-β produced by tumors inhibits dendritic cell migration from the tumor site to lymphatic drainage, increasing the risk of tumor metastasis (131) (**Figure 1**).

#### 3.3.3 RANK-RANKL and dendritic cells

The RANK-RANKL axis is closely related to DC. RANKL was first identified in 1997 using human bone marrow-derived DC (132), and RANK signaling in DC leads to immune tolerance in many cases (132–135). For example, RANKL from tumors of the genital tract induces an immature and tolerogenic phenotype in DC (136). DC are critical in antitumor combination therapies with anti-CTLA-4 and anti-RANKL antibodies (137). Thus, RANK signaling in DC may contribute to immune tolerance in bone metastatic cancers. RANKL produced by pDC can directly affect MDSCs by inducing their differentiation into osteoclasts, which promotes bone destruction and growth of breast cancer cells (138) (**Figure 1**). Furthermore, infiltration of pDC in cancer is associated with elevated levels of chemokines and cytokines that are directly or indirectly related to immunosuppression and osteoclastogenesis (138). These soluble factors also induce RANKL expression, which further stimulates osteoclastogenesis.

In conclusion, the dual role of DC in metastatic bone cancer is important for understanding the dynamic balance of the immune microenvironment and the metastatic mechanism of tumors. These findings are expected to provide new ideas for future immunotherapeutic strategies to improve immune system control of bone metastatic cancer.

### 3.4 Megakaryocytes

Megakaryocytes (MKs) are a class of cells derived from bone marrow-resident hematopoietic stem cells (HSC), which play a role in platelet production by responding to thrombopoietin (TPO) and exert a regulatory role in platelet production through their response to TPO. In addition to their effects on platelet production, MKs also affect osteoclasts and osteoblasts, thus regulating the bone microenvironment (139–142). Therefore, MKs also play a key role in bone metastatic cancer. MKs can inhibit osteoclast function while promoting osteoblast proliferation (143). In a mouse model, intracardiac injection of TPO-treated prostate cancer cells reduced the formation of bone metastases (144). Furthermore, the number of MKs in the bone marrow increased after intracardiac injection of highly osteogenic breast cancer cells (145, 146), suggesting that MKs play a key role in bone metastasis.

## 4 Osteoclasts and osteoblasts in bone metastatic cancer

### 4.1 Osteoclasts

Osteoclasts are a specialized class of cell types derived from monocytic macrophages (147). Their development and function are regulated, in part, by CSF-1 and RANKL (148). RANKL and CSF-1 bind to RANK in mature osteoclasts to induce the process of bone resorption. Furthermore, the balance between RANKL and its osteoprotegerin receptor (OPG) plays a key role in the regulation of osteoclast function. Knockdown of OPG in mice resulted in a decrease in bone density, while overexpression of OPG increased bone density (149).

During osteolytic bone metastasis, tumor cells continuously secrete a variety of osteoclastogenic cytokines in the bone, including CSF-1, PTHrP, RANKL, IL-8, IL-11, prostaglandin E, matrix metalloproteinase 1 (MMP-1), stromal cell communication network (CCN), and TNF-α (150–156). These factors directly stimulate osteoclast-mediated bone resorption and lead to the release of bone-derived tumor growth factors such as TGF-β, insulin-like growth factors (IGF), platelet-derived growth factor (PDGF), and bone morphogenetic protein (BMP) in the bone matrix, which promotes tumor growth in bone metastases (157, 158). TGF-β, a factor released after bone matrix lysis, stimulates the tumor’s secretion of PTHrP directly (159). This osteolytic cascade response is driven by the production of PTHrP. PTHrP plays a dual role in bone reconstruction. First, PTHrP upregulates monocyte chemoattractant protein-1(MCP-1) in osteoblasts, a key mediator of osteoclastogenesis, leading to the formation of osteoblastic lesions (160). Second, PTHrP stimulates osteoclast formation by improving osteoblast production of RANKL and CCL2 (142). Thus, a mutual promotion between tumor cells and osteoblasts, in which tumor cells promote osteolysis, and osteolysis then releases tumor growth factors that promote tumor growth, becomes a key therapeutic challenge. Breast cancer cells secrete an integrin-binding sialoprotein (IBSP) in bone, which attracts osteoclasts and creates an osteoclast-rich bone microenvironment (161). The R-responsive protein 2 (RSPO2) ligand in breast cancer cells interacts with RANK to promote osteoclast-mediated osteolysis (162). Furthermore, an ATP-dependent transporter protein called ABCC5 also mediates osteoclast-mediated bone resorption in breast cancer bone metastases (163). Inhibition of AEPase activity in breast cancer cells reduces osteoclast differentiation while attenuating osteolytic lesions caused by breast cancer bone metastases from breast cancer (164). Furthermore, early growth response-1 (EGR1) plays a direct role in the regulation of angiogenesis and osteoclastogenic factors in prostate cancer bone metastasis (165). Induction of tumor cells stimulates osteoclasts to secrete the IL-20RB ligand IL-19, which activates JAK1/STAT3 signaling and thus promotes proliferation of bone metastatic cancer cells (166). Furthermore, galactose lectin-3 (Gal-3) is located on the surface of osteoclasts and regulates the microenvironment of osteolytic bone metastatic cancer in the presence of RANKL (167). In bone metastatic cancers, CD47 on the surface of multiple cells regulates osteoclasts by modulating nitric oxide synthase activity, thus increasing the risk of tumor bone metastasis (168).

Exosomes secreted by tumor cells promote osteoclast differentiation and activation, leading to bone damage and remodeling of the bone metastasis microenvironment. The exosome miR-21 from SCP28 cells promotes osteoclast formation by regulating the expression of PDCD4 protein (155). In breast cancer bone metastasis, the miR-124/IL-11 axis plays a crucial role in the survival and differentiation of osteoclast progenitor cells (169). Furthermore, osteoblastic tumor exosomes can also induce osteoclast differentiation (170). For prostate cancer cells, extracellular vesicles (EVs) promote osteoclast formation in the presence of RANKL (171). Furthermore, tumor EVs of atypical cancer origin can also promote bone metastasis in hepatocellular carcinoma (172).

The mechanical environment of the bone is also critical for bone metastatic cancer. The activities of osteoblasts and cancer cells are regulated by the mechanical environment. Early changes in the mechanical environment can activate osteoclasts, which can lead to extensive osteolytic bone loss triggered by advanced bone metastatic cancer (173).

**Figure 2:**
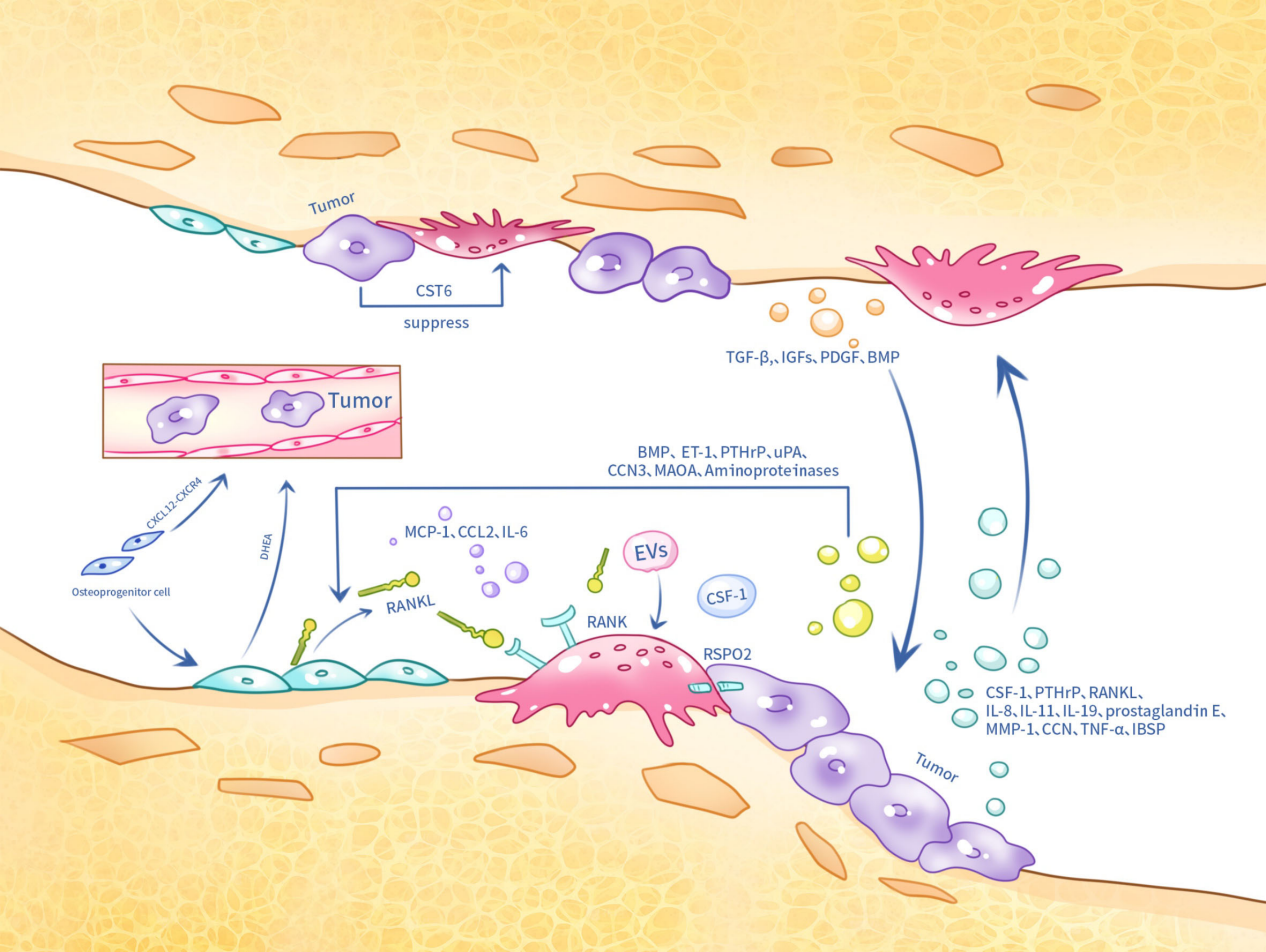
Tumor cells, osteoclasts and osteoblasts in metastatic bone cancer interact with each other at the skeletal site by several means.

However, it is important to note that tumors also exert a dual effect on osteoclasts in the bone environment. In bone metastasis, tumor cell-secreted CST6 enters osteoclasts and inhibits the activity of the cysteine protease cathepsin B (CTSB), leading to the up-regulation of sphingosinekinase1 (SPHK1), which inhibits RANKL-induced activation of p38 and suppresses osteoclast maturation (174) (**Figure 2**).

To inhibit tumor growth, some clinical strategies can be achieved by breaking this “vicious cycle”. For example, procoxacin reduces prostate cancer bone metastasis by disrupting the feedback loop of the TGF-β/C-Raf/MAPK pathway and inhibiting osteoblast and osteoclast activity (175). Denosumab is a fully human IgG2 monoclonal antibody that specifically targets RANKL. It binds to RANKL with high affinity and specificity and inhibits the binding of RANKL to osteoclast precursors and osteoclast surface RANK, thus inhibiting osteoclast differentiation and activity, and disrupting the “vicious cycle” in tumor bone metastasis. This helps to inhibit excessive bone resorption and reduce bone destruction (176).

Bisphosphonates are a class of drugs that are absorbed by bone at sites of active bone metabolism (177). Bisphosphonates inhibit osteoclast activity and survival, reducing osteoclast-mediated bone resorption. Furthermore, they can also cause osteoclast apoptosis and have a direct apoptotic effect on tumor cells (178). Therefore, bisphosphonate therapy is now the standard of care for patients with malignant bone disease in a variety of tumor types, including prostate, breast, lung, and multiple myeloma (179). Furthermore, STING agonists can also modulate osteoclast function in the TME and reduce osteolysis, thus slowing tumor progression (180).

### 4.2 Osteoblasts

Osteoblasts are derived from mesenchymal stem cells whose primary function is bone formation. The Wnt and Runt-related transcription factor 2 (Runx2) pathways play a key role in the maturation and directed differentiation of osteoblasts (181). A hallmark of osteoblast differentiation is the formation of type 1 collagen, and this process becomes critical when mediated by Shh signaling directed at prostate cancer. The stromal collagen and Shh signaling pathways act synergistically and are essential for osteoblast formation. Although cancer bone metastasis is generally presented as osteolytic metastasis, the main mechanism of cancer bone metastasis is osteogenic metastasis (182).

Prostate cancers secrete a variety of factors, such as BMP and endothelin-1 (ET-1), which promote the maturation of osteogenic precursor cells, PTHrP, which inhibits osteoblast apoptosis, aminoproteinases, which indirectly promote bone formation, and urinary fibrinogen activator (uPA) (183). These conditions contribute to the enhanced deposition of a new bone matrix. Furthermore, prostate cancer secreted CCN3 improves the expression of BMP, Runx2, and osterix in osteoblasts through glycogen synthase kinase3β (GSK3β) and β-catenin signaling pathways (184).

Although cancer bone metastasis is predominantly osteolytic, CD137 has been reported to recruit monocytes/macrophages to migrate into the TME and promote the differentiation of monocytes or macrophages into osteoblasts during bone metastasis (89). Hypoxic conditions activate HIF-1α, a specific signaling factor for osteoblasts. Activation of HIF-1α signaling increases CXCL12 blood levels, which directly activates the CXCR4 receptor and promotes the migration of breast cancer cells to bone (185).

Furthermore, in clinical practice, the reduction of androgen levels is one of the main approaches in prostate cancer treatment. Osteoblasts have been reported to secrete the adrenal androgen precursor dehydroepiandrosterone (DHEA), which does not induce the androgen receptor (AR), but promotes cancer progression and metastasis (186). Monoamine oxidase A (MAOA) also plays an important role in prostate cancer bone metastasis. MAOA stimulates the release of IL-6 from osteoblasts, which creates a bone microenvironment conducive to the homing, growth and survival of cancer cells, and also activates osteoclastogenesis through the production of RANKL and IL-6 by osteoblasts, which contributes to the development of bone metastases from cancer (187). Furthermore, VCAM1 has been reported to activate painless micrometastases by recruiting osteoblast progenitor cells (188). Studies in animal models have shown that Plumbagin successfully inhibited breast cancer cell metastasis and osteolysis by significantly altering the RANKL/OPG ratio in osteoblasts (189) (**Figure 2**).

## 5 Tumor dormancy in tumor metastasis to bone

Tumor metastasis formation is a complex process that includes local invasion and infiltration of tumor cells, survival, and extravasation of tumor cells after entering the circulation, as well as survival and proliferation in target organs (190). After invading the bloodstream, tumor cells are defined circulating tumor cells (CTCs) (191). A small percentage of tumor cells can reach distant organs to colonize (192). Once they reach a distant site, tumor cells remain dormant until that environment can support tumor growth and proliferation (193, 194). Numerous clinical studies have found that tumors have metastasized to bone early in their development, entering a dormant state in preparation for future growth (195). Clinical observations have found that for tumors that are susceptible to bone metastasis, the number of patients with skeletal lesions is less than the number of patients with diffuse tumor cells (DTC) detectable in the bone marrow, a finding that supports the idea that the bone microenvironment supports tumor dormancy (196, 197). Therefore, understanding the relationship between the bone microenvironment and tumor dormancy can contribute to subsequent treatment and research.

### 5.1 Metastasis

Perivascular cells highly expressing CXCL12 in vascular microhabitats in bone marrow sinuses can keep breast cancer cells dormant in the vasculature through CXCL-12/CXCR4 interaction (198). Immunohistochemical analysis of bone marrow from breast cancer patients showed that dormant breast cancer cells preferentially localize in CXCL12-rich vascular regions (198). CXCR4/CXCL12 also plays a crucial role in bone metastasis of prostate cancer (199). However, unlike breast cancer, prostate cancer cells may benefit from this supportive environment that maintains dormancy, but does not contribute to tumor growth (193). Furthermore, growth-arrest specific 6 (GAS6) can induce tumor dormancy in cancer (200).

### 5.2 Influence of immune factors

The bone microenvironment is also an immune-privileged site that protects dormant tumor cells from environmental damage and resulting immune responses. Tregs in the bone immune microenvironment can create an immune microenvironment that supports the growth of dormant tumor cells, allowing them to evade immune attacks (201). MDSCs in the bone microenvironment can prevent the removal of dormant tumor cells by inhibiting the activity of anti-TME CTLs and NK cells (10); Furthermore, bone marrow mesenchymal stem cells can also protect dormant tumor cells (195).

## 6 Immunotherapy for metastatic bone cancer

Bone metastatic cancers are resistant to a variety of immunotherapies due to a specific immunosuppressive microenvironment (10, 202). As a result, current treatment for patients with bone metastases has focused primarily on palliative therapies to reduce pain and improve quality of life. Due to the specificity and importance of multiple immune cells in bone metastatic cancer, it is particularly crucial to find effective immunotherapy methods for bone metastatic cancer.

Human PD-1 (CD279), encoded by the PDCD1 gene, is a transmembrane protein that is expressed as an immunosuppressive receptor predominantly on monocytes, B cells, NK cells, macrophages, and activated T-cells (203–206). Its ligands PD-L1 and PD-L2 are expressed in DC, macrophages, and tumor cells (207, 208). PD-1 activation can mediate T cell inactivation and block signaling downstream of T-cell receptor (TCR) activation (209, 210). In immunotherapy, α-PD-1 drugs, such as nivolumab, can bind to immune cells such as T cells, B cells, NK cells, macrophages, and monocytes expressing PD-1, thus blocking PD-1 signaling (203–205). This helps to keep T cells continuously activated to fight off tumors. In patients with bone metastases from non-small cell lung cancer, overall survival increased by 7.9 months in patients with nivolumab (211), suggesting that α-PD-1 therapy may be useful to reduce tumor burden in patients with bone metastases.

Combining a PD-1 blocker (nivolumab) with a CTLA-4 blocker (ipilimumab) is more effective than PD-1 blockers alone and is a standard of care for many different cancers (211). In a study of advanced renal clear cell carcinoma, a lower 12 month OS rate was found in patients with bone metastases treated with ipilimumab/nivolumab (41.7%) compared to patients without bone metastases (82.7%) (212). Another retrospective study of patients with renal cell carcinoma (RCC) bone metastases treated with ipilimumab/nivolumab found relatively low efficacy (21%) and median OS (25.6 months) (213), and, generally, patients with bone metastases responded poorly to the combination of PD-1 and CTLA-4 inhibitors (213, 214). This may be related to the fact that bone metastatic cancers present an immunologically “cold” phenotype (215) and an immunosuppressive microenvironment (including infiltration of multiple immunosuppressive cells such as Tregs). Thus, eliminating Tregs in the bone metastatic microenvironment is a promising aspect of immunotherapy. Furthermore, anti-PD-1 immunotherapy can also produce long-term benefits in preventing bone destruction and relieving pain in bone cancer by inhibiting osteoclastogenesis (216).

NK cells kill tumors through multiple mechanisms, including granzyme B and perforin-mediated apoptosis or Fas-Fas ligand interactions. When IRF7 levels are restored, the response of host NK cells can be reactivated (113). Furthermore, gut microbial supplementation also helps promote NK cell proliferation in metastatic bone tumors (17). The ability of modified NK cells to produce IL-2 and IL-15, stimulate proliferation, and increase resistance to tumors makes them a new option for the treatment of bone metastatic cancer (217).

Macrophages play a crucial role in the immunotherapy of bone metastatic cancer.M2-type macrophages inhibit CD8+ T-cell resistance to tumors through multiple pathways. The use of anti-CD115 antibodies, trabectedin, clodronic acid, and zoledronic acid reduces the number of macrophages within the tumor (218). Macrophages are recruited to tumor sites primarily through the CCL2/CCR2 axis and CSF-1/CSF-1R signaling, so blocking these two signaling axes also reduces macrophage infiltration (219–221).

MDSCs are extensively infiltrated in metastatic bone cancers and have a significant ability to suppress T cell responses. Based on available evidence, the use of CXCR4 antagonists and indoleamine2,3-dioxygenase1 (IDO1) inhibitors activates CD8+ T cells and suppresses MDSCs, thus delaying bone metastasis in mouse breast cancer disease (222). Dickkopf-1 (Dkk1), a secreted Wnt antagonist, modulates the number and function of MDSCs in bone metastases in mice (223). Furthermore, multiple chemokine axes, such as CCR2/CCL2, CXCR2/CXCL5, and CXCR4/CXCL12, and inhibition of these signaling pathways prevents the entry of MDSCs from the bone marrow into the TME (10).

In immunotherapy, tolerogenic DC or pDC in tumors can affect the killing function of CD8+ T cells. By using PDCA1 antibodies, pDC can be reduced, thus reducing the load of bone metastases in breast cancer (128). Furthermore, microtubule destabilizers (e.g., dolastatin 10 and ansamitocin P3) can convert tolerant DC into activated DC that stimulate the killing effect of CD8+ T cells, which in turn fight the tumor (224). DC vaccines have also been considered a new approach to treating bone metastases by injecting DC-carrying tumor antigens to activate the immune response within the tumor (38). In a model of melanoma metastasis, stimulation of DC with cyclic VHCDR3-derived peptide (Rb9) inhibited melanoma metastasis (225). CD103+cDC1 vaccine inhibited primary and metastatic tumor growth, and IL-12 produced by CD103+DC was critical for NK cell-mediated tumor control (226, 227) (**Table 1**).

**Table 1:** The latest treatment for several immune cells.

Cell Type	Treatment
CD8+T	α-PD-1,α-CTLA-4
NK cell	α-PD-1,IRF7 Agonists,gut microbe,Engineered NK cells(IL-2,IL-15)
macrophage	α-PD-1, anti-CD115 antibodies,trabectedin,clodronic acid,zoledronic acid,CCR2 inhibitors,CSF1R inhibitors
MDSCs	CXCR4 antagonists,CCR2 inhibitors, CXCR2 inhibitors,IDO1 inhibitors,Dickkopf-1
DC	PDCA1 antibodies,DC vaccines,microtubule destabilizers,VHCDR3-derived peptide
osteoclast	Procoxacin anti-RANKL antagonists. Bisphosphonates. STING agonists
osteoblast	Procoxacin. Plumbagin

## 7 Discussion

Survival of patients with multiple solid tumors that metastasize to the bone is a great challenge. Previous studies have thoroughly explained the “vicious cycle” between tumor cells that metastasize to bone and osteoclasts and osteoblasts, and there are various therapeutic approaches, including the use of deslumab and bisphosphonates, that can break the “vicious cycle”. Meanwhile, the role of various types of immune cells and non-immune cells in the bone microenvironment during the transfer of tumor cells from the primary site to the bone has also been well studied. However, the multiple effects of multiple immune cells on adaptive immunity, i.e., on the specific tumor-killing effects of CTLs, after tumor cells colonize the immune microenvironment following bone are still not well reviewed, and thus a better understanding of the immune microenvironment of metastatic bone cancer is crucial for multiple effects. How to balance the immune cell effects on tumor-killing function and tumor growth promotion is a central question for the subsequent exploration of therapeutic approaches for bone metastatic cancer. Breaking the suppressive function of immune cells on adaptive immunity and enhancing the tumor-killing effect of immune cells on promoting CTLs will be the direction of future research on the immune microenvironment of bone metastatic cancer. In this review, we describe the multiple effects of various immune cells in the bone immune microenvironment, including osteoclasts and osteoblasts, on tumor metastasis and on adaptive immunity, highlighting the specific mechanisms by which the various types of immune cells function.

We also discuss tumor dormancy at the skeletal site, including the various types of immune factors that may influence tumor dormancy. Many solid tumors develop bone metastases at an early stage, but the tumor cells are dormant and the bone microenvironment protects the dormant tumor cells. Studying the effects of immune cells in the bone microenvironment on dormant tumor cells can guide clinical treatment for preventing bone metastasis in solid tumors. How to kill dormant tumor cells while avoiding harmful effects on the body’s normal bone immune microenvironment is still a question that needs to be explored.

We also reviewed current and future therapeutic approaches for the treatment of bone metastatic cancers. Within conventional immunotherapeutic agents, α-PD-1 agents have been shown to be helpful in reducing the tumor burden in patients with bone metastases from non-small cell lung cancers, and because of the special microenvironment of bone, α-PD-1 immunotherapy also has an impact on other factors such as osteoclasts, making the future of α-PD-1 in bone metastatic cancers also worthy of explore., as our understanding of the signaling mechanisms between tumor cells and cells in the bone immune microenvironment increases, several emerging therapeutic approaches, such as modification of NK cells, targeting of MDSCs and macrophages, and DC vaccines, can also be effective and efficient in halting the progression of skeletal lesions.

In conclusion, the interplay between the intrinsic cells of the bone, the immune cells, the bone matrix, and the tumor cells is critical for the progression of the tumor. Once the tumor invades the bone, how to prevent the immune cells from being called “accomplices” of tumor progression is still a question. What factors can break the “vicious cycle” between the four also needs to be further investigated. What factors promote tumor dormancy in metastatic bone cancer, and what factors cause dormant tumor cells to awaken and proliferate. Traditional immunotherapy is not effective in metastatic bone cancer, and it is worth exploring how to improve the effectiveness of immunotherapy in metastatic bone cancer by targeting various types of immune cells. The future of many emerging therapies is bright, but further research is needed to exploit the specificities of the bone microenvironment to combat bone tumors.

## Author contributions

CS: Writing – original draft, Writing – review & editing. LJ: Writing – original draft, Writing – review & editing. MH: Writing – original draft, Writing – review & editing. ZW: Writing – original draft. JL: Writing – original draft. LS: Writing – review & editing. YE: Writing – review & editing. XY: Writing – original draft, Writing – review & editing. ZS: Writing – review & editing. TC: Writing – review & editing. WF: Writing – review & editing. ZS: Writing – review & editing. CX: Writing – review & editing. WZ: Writing – original draft. ZJ: Writing – original draft. ZZ: Writing – original draft. ZY: Writing – review & editing. LB: Methodology, Resources, Supervision, Writing – original draft, Writing – review & editing.
